# Binding and Detoxification of Insecticides by Potentially Probiotic Lactic Acid Bacteria Isolated from Honeybee (*Apis mellifera* L.) Environment—An In Vitro Study

**DOI:** 10.3390/cells11233743

**Published:** 2022-11-23

**Authors:** Aleksandra Leska, Adriana Nowak, Karolina Miśkiewicz, Justyna Rosicka-Kaczmarek

**Affiliations:** 1 Department of Environmental Biotechnology, Faculty of Biotechnology and Food Sciences, Lodz University of Technology, Wolczanska 171/173, 90-530 Lodz, Poland; 2 Institute of Food Technology and Analysis, Faculty of Biotechnology and Food Sciences, Lodz University of Technology, Stefanowskiego 2/22, 90-537 Lodz, Poland

**Keywords:** insecticides, probiotics, detoxification, *Apis mellifera* L., toxicity, imidacloprid, coumaphos, chlorpyrifos, Sf-9, Caco-2

## Abstract

Lactic acid bacteria (LAB) naturally inhabiting the digestive tract of honeybees are known for their ability to detoxify xenobiotics. The effect of chlorpyrifos, coumaphos, and imidacloprid on the growth of LAB strains was tested. All strains showed high resistance to these insecticides. Subsequently, the insecticide binding ability of LAB was investigated. Coumaphos and chlorpyrifos were bound to the greatest extent (up to approx. 64%), and imidacloprid to a much weaker extent (up to approx. 36%). The insecticides were detected in extra- and intracellular extracts of the bacterial cell wall. The ability of selected LAB to reduce the cyto- and genotoxicity of insecticides was tested on two normal (ovarian insect Sf-9 and rat intestinal IEC-6) cell lines and one cancer (human intestinal Caco-2) cell line. All strains exhibited various levels of reduction in the cyto- and genotoxicity of tested insecticides. It seems that coumaphos was detoxified most potently. The detoxification abilities depended on the insecticide, LAB strain, and cell line. The detoxification of insecticides in the organisms of honeybees may reduce the likelihood of the penetration of these toxins into honeybee products consumed by humans and may contribute to the improvement of the condition in apiaries and honeybee health.

## 1. Introduction

Pesticides are natural or synthetic chemicals used to control crops’ diseases, weeds, and pests. These chemical substances comprise a wide range of insecticides, herbicides, fungicides, etc. [1]. Over the past few decades, pesticides have played an important role in improving agricultural productivity. In modern agriculture, pesticides are used to increase yield quantity and quality. However, due to their extensive use, there is a risk of some residues being present in the environment for many years [2]. Pesticides contained in crops, soil, air, and water enter the food chain, posing a serious threat to living organisms. Furthermore, pesticides can enter the bodies of animals and humans through various routes, such as ingestion, inhalation, or absorption through damaged skin, and then be metabolized and bioaccumulated in body fat [3]. Pesticides also disturb the soil ecosystem and negatively affect the microbial diversity and fertility of agricultural lands by reducing seed quality [4]. The vast majority of pesticides that are used commercially consist of active and other additional ingredients that are added to increase their effectiveness. The exact compositions are often unknown and protected by producers [5].

Honeybees (*Apis mellifera* L.) are exposed to frequent contact with a wide variety of pesticides due to their versatile use in the modern world. A number of pesticides have sublethal effects on honeybees, causing shortened adult longevity, immune system impairment, and delayed development [6]. Pesticides also have a negative effect on the reproduction of honeybees [7]. For example, the residues accumulated in beeswax reduce the number of eggs laid and the ovarian weight of queens [7]. Adult honeybees are more exposed to pesticides through constant contact with contaminated nectar and pollen; however, they can carry chemical contaminants to the hives, putting younger individuals at risk [8]. Unlike other insects, the honeybee genome contains fewer genes encoding enzymes associated with insecticide resistance such as carboxyl/cholinesterases, glutathione-s-transferases, and cytochrome P450 monooxygenases [9]. Due to deficiencies in these enzymes, honeybees are highly vulnerable to the negative effects of pesticides. Of the global pesticide consumption, 44% is targeted to kill insects [4]. Insecticides endanger honeybee health in various ways, e.g., by directly killing individuals through acute toxicity [10]. Honeybees are most at risk of primary exposure through contact with flowers sprayed immediately after application. A well-known insecticide that threatens honeybees is imidacloprid, which belongs to the group of neonicotinoids [11]. Imidacloprid negatively affects the central nervous system of insects by interfering with the acetylcholine receptors (AchR) responsible for neurotransmission [12]. Honeybees exposed to this chemical exhibit reduced resistance to pathogenic microorganisms and antibiotics, resulting in increased mortality in the colony [13]. Moreover, imidacloprid interferes with the abilities of honeybees to forage and learn and remember food source locations [14]. Another insecticide that disrupts the memory of honeybees is chlorpyrifos. Sublethal doses of chlorpyrifos can threaten the survival of honeybees by weakening their ability to learn, which plays an important role in communication and behavioral ecology [15]. The toxicity of chlorpyrifos decreases three days after application to crops, although the negative effects of the exposure are still noticeable [16]. Chlorpyrifos has been phased out in many countries, but its residues are detectable in honey and honeybee products due to its persistence in the environment [17,18]. An example of another organophosphate pesticide with ectoparasiticide properties is coumaphos [19]. Coumaphos can reduce the level of honeybee gene products related to hormonal and cellular immunity and disrupt the detoxification processes of chemical substances. It also affects the physiological functions and immune responses of honeybees, making them vulnerable to diseases caused by pathogens [20]. Honeybee health is largely dependent on digestive tract microbiota, which contribute to the host’s homeostasis. Environmental stressors such as pesticides can affect the insect holobiont, leading to a weakened immune system [21,22,23]. Insecticides can alter the functional potential of microbiota, increasing colony mortality by disrupting processes such as oxidative phosphorylation or sugar metabolism [24]. Some pesticides target enzymes present in almost all sequenced genomes of honeybee gut bacteria, indicating their potential susceptibility [25]. Pesticides such as acetamiprid, oxalic acid, and thiacloprid completely alter the honeybees’ gut microbiota pattern compared to that of the control honeybees [26]. For example, oxalic acid caused a decrease in the number of bacteria from the genera *Bartonella* and *Klebsiella* and an increase in those from the genera *Bifidobacterium* and *Gilliamella* and elimination of the genus *Bombella* [26]. Oxalic acid also disturbed the pH of the digestive tract. In general, pesticides lead to an imbalance in the microbiome of the honeybees’ digestive tract, thus, causing changes in the composition, biodiversity, and physiology of their natural microbiome, leading to dysbiosis. The result can be metabolic changes, an imbalance in the proportions between different microbial taxa, disruption of the detoxification system, weakened immunity, and increased susceptibility to infection by pathogens and opportunistic pathogens [21,22,23]. 

Lactic acid bacteria (LAB) are natural inhabitants of the digestive tract of honeybees which perform numerous beneficial functions in the body [27]. Honeybee microbiota play an important role in detoxifying various xenobiotics, however, LAB inhabiting the digestive tract of honeybees exhibit high sensitivity to chemical substances [26,28]. In recent decades, probiotics have gained attention for their extensive detoxification properties [29]. LAB degrade and absorb microbial toxins, bind heavy metals such as inorganic (Hg(II)) and organic (CH_3_Hg) forms of mercury, and hydrolyze toxic chemicals to carbon sources [30,31]. Certain LAB cell wall components such as peptidoglycans and polysaccharides have been proven to bind specific toxins [32]. According to Nowak and Libudzisz, the ability of LAB to bind certain toxic compounds such as N-nitrosodimethylamine (NDMA) depends on the concentration, the time of incubation, and the culture medium [33]. Some LAB strains show the potential to remove carcinogenic compounds such as heterocyclic aromatic amines, mycotoxins, or benzopyrene through cell wall absorption [34,35,36,37]. The inactivation of mutagens is related to the antigenotoxic activity of LAB and detoxification of the medium’s environment from substances that endanger the health of the host [38].

Exposure to pesticides can also have negative effects on human cells. Some insecticides increase cell death, cause significant peroxidation of cells’ lipids, and destroy membranes of human immune cells [39,40]. Exposure to pesticides is associated with numerous negative dermatological, gastrointestinal, carcinogenic, respiratory, reproductive, and neurological effects [41]. An example is a rare idiosyncratic bone marrow reaction to pesticides which probably leads to aplastic anemia [42]. Exposure to pesticides also leads to chromosomal damage and various neurological diseases. An example is organophosphorus compounds, which, in addition to their acute neurological effects, are associated with psychological effects (e.g., behavioral changes) [43]. Knowing this, LAB should be able to detoxify pesticides already in the organisms of honeybees so that lower concentrations are transferred to the honeybee products and, consequently, to the human body.

Several LAB genera are widely used as probiotics, classified as “Generally Recognized as Safe (GRAS)” by the European Food Safety Authority (EFSA) [44]. Some LAB strains can subvert the sublethal effects of pesticides and help to improve the long-term survival of honeybees [45]. Strengthening the microbiota of these pollinators would reduce mortality in colonies and reduce the concentration of pesticides contained in honeybee products. Until this date, knowledge of the detoxification abilities and insecticide resistance exhibited by LAB has been very limited. 

Our study focused on determining the pesticide detoxification capacity displayed by potentially probiotic LAB strains that were mostly isolated from honeybee environments. The potential of pesticide detoxification by LAB strains present in the honeybee environment has not been confirmed. There is also a lack of in-depth research on the effect of LAB on the reduction of cytotoxicity and genotoxicity of pesticides in cell lines. Thus, the aim of the present study was to evaluate the ability of several LAB strains, mostly isolated from honeybee environments [46], to detoxify three insecticides: chlorpyrifos, coumaphos, and imidacloprid. In the first stage, the survival of LAB in the presence of the above-mentioned insecticides was studied, followed by the ability to bind them to the bacterial cell wall (by live and thermally inactivated cells), and then the degree of their detoxification (cytotoxicity and genotoxicity) was estimated on three cell lines.

Due to the detoxification abilities of LAB, it is worthwhile to enrich the microbiota of honeybees with preparations containing selected probiotic strains with such properties. The results of this research may lead to the finding of LAB strains with the strongest ability to detoxify the tested pesticides and which, in the future, could be used to construct a probiotic protective preparation for honeybees that could protect these insects from the negative effects of pesticides while contributing to the reduction of pesticide residues in honey and honeybee products.

## 2. Materials and Methods

### 2.1. Culture Vessels, Chemicals, and Other Materials

Phosphate buffer saline (PBS), deMan–Rogosa–Sharp (MRS) broth, glass beads, high-glucose and low-glucose Dulbecco’s Modified Eagle Medium (DMEM), RPMI 1640, 4-(2-hydroxyethyl)-1-piperazineethanesulphonic acid (HEPES), streptomycin–penicillin mixture for cell cultures, insulin, fructose, cysteine hydrochloride, hydrogen peroxide (H_2_O_2_), LMP (low melting point) and NMP (normal melting point) agaroses, sodium chloride (NaCl), Triton X-100, EDTA, Tris, sodium hydroxide (NaOH), 3-(4,5-dimethylthiazol-2-yl)-2,5-diphenyltetrazolium bromide (MTT), 4′,6-diamidino-2-phenylindole (DAPI), dimethyl sulfoxide (DMSO), coumaphos, chlorpyrifos, imidacloprid, and trypan blue were purchased from Merck Life Science, Warsaw, Poland. Fetal bovine serum (FBS), GlutaMAX^TM^, TrypLE^TM^ Express, Sf-9 cell line, Sf-900™ III Serum-Free Medium (SFM), and AnaeroGen Atmosphere Generation Systems sachets were purchased from Thermo Fisher Scientific, Waltham, MA, USA. Cryobanks™ were from Copan Diagnostics Inc., Jefferson Avenue Murrieta, Murrieta, CA, USA. Organic solvents such as acetonitrile (HPLC grade) were from J.T. Baker (New York, USA). Ultra-purified water (resistivity 18.2 MΩ cm^−1^) was obtained using Milli-Q Plus Technology, Millipore (Bedford, MA, USA). The Caco-2 cell line was from Cell Line Service GmbH, Eppelheim, Germany, while the IEC-6 cell line was purchased from DSMZ German Collection of Microorganisms and Cell Cultures GmbH, Braunschweig, Germany. In addition, 96-well plates, serological pipettes, and roux flasks T25 and T75 (all from Greiner Bio-One GmbH Kremsmünster, Austria) were purchased from Biokom Systems, Janki, Poland. Syringe filters (0.22 µm and 0.45 µm pore size) were purchased from Labindex S.A., Warsaw, Poland.

### 2.2. Lactic Acid Bacteria Strains and Growth Conditions

A total of 25 strains of LAB were used for this study. They were selected based on previous research, i.e., those with the strongest antagonistic activity towards honeybee pathogens, such as *Paenibacillus larvae* or *Melissococcus plutonius* [47], and characterized by high adherence abilities (unpublished data). These were: *Lactiplantibacillus plantarum* (8AN, 145, 10/2, 14/3, 18/1, 21/1), *Pediococcus acidilactici* (4/1, 5/2, 6/1, 7/1, 8/1, 9/3, 22/1, 25/1, 35/1), *Pediococcus pentosaceus* (11/3, 14/1, 19/1), *Levilactobacillus brevis* KKA, *Lacticaseibacillus casei* 12AN, *Lactobacillus acidophilus* 573, *Ligilactobacillus salivarius* 9AN, and *Pediococcus parvulus* OK-S. These were isolates from honeybee environments (i.e., *L. plantarum* 10/2, 14/3, 18/1, and 21/1; *P. acidilactici* 4/1, 5/2, 6/1, 7/1, 8/1, 9/3, 22/1, 25/1, and 35/1; *P. pentosaceus* 11/3, 14/1, and 19/1) such as flowers, honey, or bee pollen (their isolation and characteristics were published previously [46]), as well as collection strains (*L. plantarum* 8AN and 145; *L. brevis* KKA; *L. casei* 12AN, *L. acidophilus* 573, *L. salivarius* 9AN, and *P. parvulus* OK-S of different origins, e.g., fermented cabbage, fermented cucumbers, human feces—see Section 3.2) acquired from the collection of the Department of Environmental Biotechnology, Lodz University of Technology. *Apilactobacillus kunkeei* DSM 12361, which is a strain naturally inhabiting the honeybee gut microbiota, was used as a control (reference) strain and was purchased from the German Collection of Microorganisms and Cell Cultures GmbH. Additionally, the commercial probiotic strain *Lacticaseibacillus rhamnosus* GG was used in the study. All LAB were stored in Cryobanks™ at −20 °C. Before experiments, strains were activated, threefold passaged (3% inoculum), and anaerobically (AnaeroGen Atmosphere Generation Systems sachets) cultured in MRS broth for 24 h at 37 °C. *A. kunkeei* DSM 12361 was cultured anaerobically on MRS broth with the addition of fructose (10 g/L) and 0.05% cysteine hydrochloride (MRS-F).

### 2.3. Pesticide Stocks Preparation and Storage

Coumaphos (organophosphorus insecticide), chlorpyrifos (organophosphorus pesticide), and imidacloprid (neonicotinoid insecticide) engaged in this study were of PESTANAL^®^ analytical standard purity (>99%). They were dissolved in sterile DMSO to obtain stocks (100 mg/mL). Stocks were stored at −20 °C in the dark. To avoid DMSO toxicity, the final concentration of DMSO in all experiments was ≤0.5%.

### 2.4. Effect of Pesticides on the Growth of LAB

The experiments were conducted in transparent 96-well polystyrene microplates. The final concentrations of tested pesticides were 20, 100, and 500 µg/mL. Each well was inoculated with an overnight culture of an individual LAB strain at the density of 1.8–2.4 × 10^9^ CFU/mL (6.0–8.0 according to McFarland standard). Each strain was tested in 4 replicates. Negative controls were non-exposed bacterial cells in MRS or MRS-F broth. The test was performed at 30 or 37 °C for 48 h.

### 2.5. Pesticide Binding Assay

#### 2.5.1. Whole Live LAB Cells

Overnight cultures of LAB in MRS/MRS-F broth were centrifuged (3864× *g*, 15 min), and biomass was washed with sterile ultrapure water and centrifuged again. This procedure was repeated until the MRS/MRS-F broth was completely washed. Next, the biomass of whole live LAB cells was suspended in sterile ultrapure water with an individual pesticide solution (at a final concentration of 100 µg/mL, *v*/*v*) and thoroughly mixed. The density of LAB cells was 1.8–2.4 × 10^9^ CFU/mL (6.0–8.0 according to McFarland standard) [48]. The positive controls (standards) were individual pesticides without LAB strains. Negative controls were LAB strains suspended in sterile ultrapure water without pesticides. The samples were incubated anaerobically at 30 °C for 24 h with continuous orbital shaking (240 r.p.m.) (LAUDA Varioshake VS 8 OE Shaker, Dr. R. Wobser Gmbh & Co., Lauda-Königshofen, Denmark). Finally, the supernatants containing residual pesticide were collected by centrifugation (10,733× *g*, 20 min) and were filtered using sterile syringe filters (0.22 μm) and frozen in sterile vials until analysis at −20 °C. 

#### 2.5.2. Thermally Inactivated LAB Cells

The above procedure (Section 2.5.1) was performed for heat-inactivated LAB strains (100 °C, 30 min) [49]. The option was conducted only for LAB strains with the strongest pesticide binding capacity (it was 13 samples) from Section 2.5.1.

#### 2.5.3. Intracellular Extracts (ICEs) and Membrane Extracts (MEs)

The remaining biomass of LAB strains (Section 2.5.1) was washed with ultrapure water and centrifuged (9279× *g*, 20 min). To prepare ICEs, ultrapure water was added to the remaining pellets. After thoroughly mixing with glass beads, samples were sonicated in an ice bath (5 min, amplitude 50, pulse 6 s, pause 2 s) with an ultrasonic homogenizer (Hielscher Ultrasonics GmbH, Germany). Then, ICEs were centrifuged (9279× *g*, 20 min), and supernatants were filtered (0.22 μm). To achieve MEs, ultrapure water was added to the remaining pellets, and, after thorough shaking for several minutes, they were centrifuged (9279× *g*, 20 min) and filtered (0.22 μm). All samples were frozen at −20 °C until analysis. The option was conducted only for LAB strains from Section 2.5.1 (it was 26 samples) with at least 30% pesticide binding capacity. 

### 2.6. HPLC Analysis

Pesticides were analyzed according to Hafeez et al. (2015) using the HPLC-DAD technique with some modifications [50]. For the qualitative–quantitative determination of pesticides, HPLC analysis was performed using a UHPLC+ Dionex UltiMate 3000 system (Thermo Fisher Scientific Inc., Waltham, MA, USA) equipped with a UV–Vis detector (Thermo Fisher Scientific Inc., Waltham, MA, USA) and NUCLEOSIL C18 column (4.6 × 250 mm, 5 µm particle size; Macherey-Nagel, Germany). Isocratic elution was carried out with 70/30 (*v*/*v*) acetonitrile/water as the mobile phase. The flow rate was set to 1.0 mL/min, and the column temperature was 25 °C. The injection volume was 20 µL. Detection and quantification were performed with two wavelengths: 219 nm for chlorpyrifos and coumaphos and 270 nm for imidacloprid. Quantification was performed using an external standard method. All measurements were made twice.

### 2.7. Cell Line Cultures (Sf-9, Caco-2, and IEC-6)

*Spodoptera frugiperda* (Sf-9, fall armyworm) insect cell line from pupa ovarian tissues was cultured as a monolayer in ready-to-use Sf-900™ III Serum-Free Medium at 27 °C in a non-humidified, ambient-air-regulated incubator (Binder BD 56, GmbH, Tuttlingen, Germany) for 10 days to reach 80% confluence. Every 2–3 days, the cells were washed with PBS (pH 7.2) without calcium and magnesium, and the medium was renewed. Confluent cells were detached from the substrate with gentle pipetting, centrifuged (200× *g*, 5 min) and decanted and then the pellet was resuspended in a fresh culture medium. 

Caco-2 (human colon adenocarcinoma) cells were cultured in high-glucose DMEM and IEC-6 (normal small intestine from rat) cells in low-glucose DMEM:RPMI 1640 (1:1, *v*/*v*) with the addition of 10% FBS, 4 mM (Caco-2) or 2 mM (IEC-6) GlutaMAX^TM^, 25 mM HEPES, 100 µg/mL streptomycin/100 IU/mL penicillin, and 0.1 U/mL insulin (IEC-6). Cells were cultured as a monolayer at 37 °C with 5% CO_2_ in a humidified incubator (Galaxy 48S, New Brunswick, United Kingdom) for 7 days to reach 80% confluence. Every 2–3 days, the cells were washed with PBS (pH 7.2) without calcium and magnesium, and the medium was renewed. Confluent cells were detached from the culture with TrypLE^TM^ Express (37 °C, 8–10 min), centrifuged (307× *g*, 5 min), and decanted and then the pellet was resuspended in a fresh culture medium. After the detaching procedure, performing a cell count by hemocytometer, and determining cell viability by trypan blue exclusion, the cells were ready to use. The viability of cells taken to the experiments was at least 80% (Sf-9) and 90% (Caco-2 and IEC-6). 

### 2.8. MTT Assay

The supernatants of LAB strains with the strongest binding capacity were taken for the experiment after the binding of pesticides (from Section 2.5.1), as well as pesticide standards (also from Section 2.5.1). The cytotoxicity was assessed with an MTT assay. A total of 5000 (Caco-2), 10,000 (IEC-6), or 20,000 (Sf-9) cells/well were placed in 96-well, transparent, flat-bottom plates and incubated for 24 h at 37 °C in 5% CO_2_ (Caco-2, IEC-6) and at 27 °C in a non-humidified incubator (Sf-9). The next day, the medium was removed and then samples and standards were added to the cells monolayer at one concentration of 20% (*v*/*v*). Each sample was tested in 4 replicates, and cells were exposed for 24 h. Cells in the control sample were exposed only to the vehicle. After the time of exposure, test samples were gently aspirated, and MTT (0.5 mg/mL in PBS) was added to each well and incubated for a further 3 h. The MTT was then aspirated, and DMSO was added to dissolve the formazan crystals. Absorbance was measured at 550 nm (using a 620 nm reference filter) in a microplate reader (TriStar^2^ LB 942, Berthold Technologies GmbH & Co. KG, Bad Wildbad, Germany). Negative control absorbance represented 100% cell viability. Cell viability (%) was calculated as: (sample OD/control OD) × 100%; and cytotoxicity (%) was calculated as: 100 − cell viability. Results are presented as the mean of the four individual readings ± standard deviation (±SD). 

### 2.9. Single-Cell Gel Electrophoresis Assay (Comet Assay)

After MTT, the samples were subjected to genotoxicity testing. The Eppendorf tubes were loaded with the appropriate cell culture medium (Caco-2, IEC-6, or Sf-9), the cells in the amount of 1 × 105 cells/sample, and the test sample so that the final volume was 1 mL. The final concentration of all test samples was 20% (*v*/*v*). Cells in the control sample were exposed only to the vehicle. Samples were then incubated for 60 min at 27 °C (Sf-9) and 37 °C (Caco-2 and IEC-6) and then centrifuged (15 min, 4 °C, 182× *g*) and decanted, and LMP agarose was added at 37 °C. The suspension was spotted on warm NMP double-layered slides and covered with coverslips (hot plate ZF6 Premiere Slide Warmer). Next, the samples were placed on a Chilling Plate for Comet Assay Slides (Cleaver Scientific, Rugby, Great Britain) and allowed to solidify. Then, alkaline lysis was performed with the buffer (2.5 M NaCl, 1% Triton X-100, 100 mM EDTA, 10 mM Tris, pH 10) and incubated (60 min, 4 °C). The lysis buffer was decanted and then the slides were flooded with the unwinding buffer (300 mM NaOH, 1 mM EDTA) (20 min, 4 °C); next, they were placed in an electrophoresis apparatus (CSL-COM20, Cleaver Scientific). The electrophoresis was performed in an electrophoretic buffer (300 mM NaOH, 1 mM EDTA, pH > 13) for 20 min at a voltage of 21 V and a current of 29 mA. The slides were neutralized and allowed to dry and then were stained for 60 min at 4 °C with DAPI (1 µg/mL); then, comet analysis was performed under a fluorescence microscope (Nikon) at a magnification of 200× equipped with a camera (Nikon Digital Sight DS-U3) and with Lucia Comet v.7.0 software (Laboratory Imaging, Prague, Czech Republic). In each trial, 50 randomly selected comets were analyzed based on the parameter determining the percentage of DNA in the comet’s “tail”. Results are presented as mean ± S.E.M.

### 2.10. Statistical Analysis

All obtained results were subjected to statistical analysis using one-way ANOVA analysis followed by Tukey’s multiple-comparisons post hoc test performed using OriginPro 6.1 (Northampton, MA, USA) software at a significance level of * *p* ≤ 0.1, ** *p* ≤ 0.05, and *** *p* ≤ 0.01. Differences between samples with normal distribution were evaluated by Student’s *t*-test.

## 3. Results and Discussion

### 3.1. LAB Growth in the Presence of Pesticides

**Figure 1:**
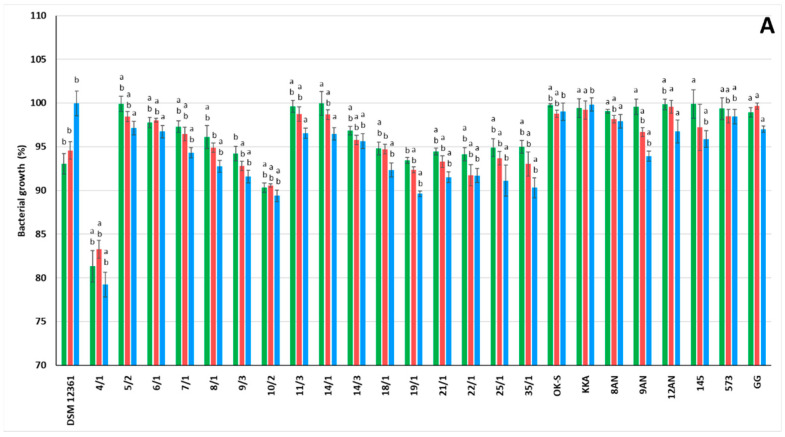

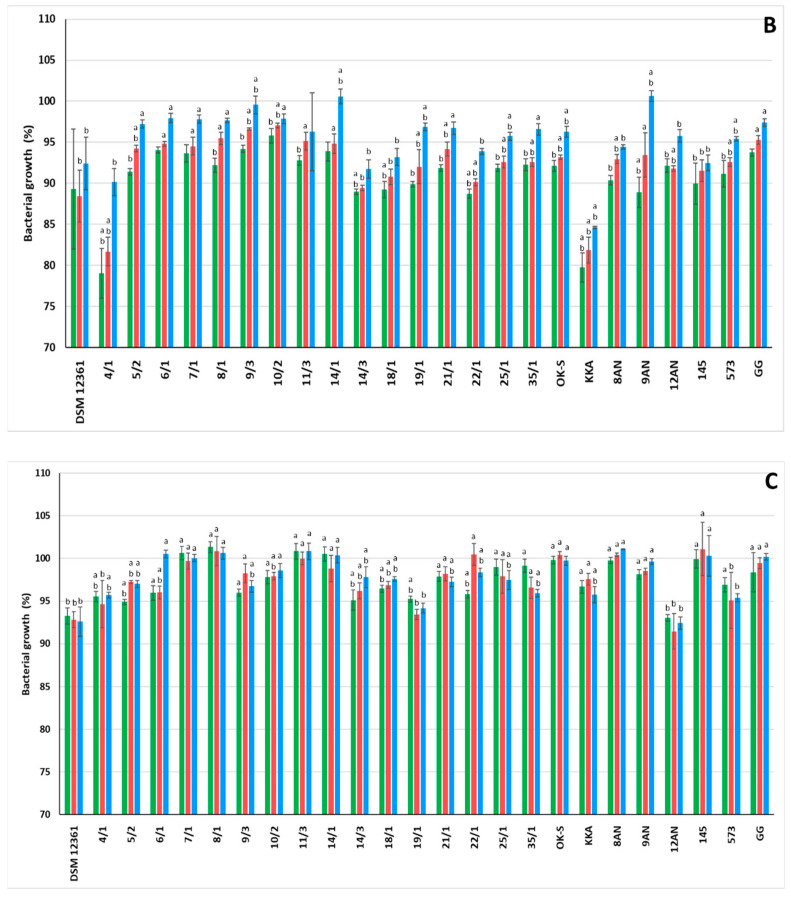
Effect of chlorpyrifos (**A**), coumaphos (**B**), and imidacloprid (**C**) on the growth of lactic acid bacteria strains during 48 h incubation, as evaluated with microtitration assay. Bars: green—20 µg/mL; red—100 µg/mL; blue—500 µg/mL. Each data point represents the mean from four individual wells. Results are presented as mean ± standard deviation (SD). Statistically significant compared to ^a^ *A. kunkeei* DSM 12361 and ^b^ *L. rhamnosus* GG at *p* ≤ 0.05.

In the study presented above, we examined the effects of three insecticides (chlorpyrifos, coumaphos, and imidacloprid) on the growth of 25 LAB strains (Figure 1). The pesticide doses chosen for the study were selected based on a literature review [51,52,53,54,55]. All tested LAB strains showed high resistance to insecticides. The obtained results suggest no significant influence of insecticide concentration on LAB growth. Collection LAB strains presented stronger resistance to chlorpyrifos; however, LAB strains isolated from the honeybee environment exhibited more potent growth in the presence of coumaphos. In the case of imidacloprid, the origin of the LAB strains did not affect their resistance to insecticides. The presence of pesticides significantly influenced the growth of the tested LAB in comparison to the control strains *A. kunkeei* DSM 12361 and *L. rhamnosus* GG (*p* ≤ 0.05). In the above study, the *P. pentosaceus* 14/1 strain showed the greatest resistance to the presence of chlorpyrifos, with bacterial growth reaching 99.94% ± 1.4 for a concentration of 20 µg/mL (Figure 1A).

Some LAB strains were able to grow even with high concentrations of chlorpyrifos (>1400 μg/mL) [51]. Honeybees come into frequent contact with chlorpyrifos due to its constant use in horticulture and agriculture. The main pathways of exposure to chlorpyrifos are through direct contact with plants sprayed with this pesticide or through pollen brought into the hive by honeybees [16]. According to Daisley et al., chlorpyrifos at a concentration of 295 µM significantly reduced the growth of *L. plantarum* ISO in MRS medium during the log phase [55]. Chlorpyrifos significantly affects the survival and shift in the gut bacterial consortium in the larvae of certain insects such as *Propsilocerus akamusi* [56]. However, there are relationships between the degradation potential of chlorpyrifos and the bacterial growth rate. In vitro tests by Yildrim Kumal et al. [57] showed that some *L. plantarum* strains metabolize this insecticide by the esterase enzyme and use these compounds as energy and carbon sources. According to Pinto et al. [58], the presence of 500 mg/L chlorpyrifos did not affect the growth of *Enterococcus faecium* E86, *Lactococcus lactis* subsp. *lactis* ATCC 11454, *L. rhamnosus* GG ATCC 53103, *L. lactis* Garvie ATCC 19256, *Leuconostoc mesenteroides* subsp. *mesenteroides* ATCC 8293, and *Pediococcus pentosaceus* Mees ATCC 43200. 

The greatest resistance to the action of coumaphos was demonstrated by the *P. pentosaceus* 14/1 strain, where the bacterial growth reached 100.6% ± 0.9 for a concentration of 500 µg/mL (Figure 1B). According to in vivo tests by Kukumanu et al. [24], coumaphos has a significant effect on the microbial community pattern of honeybees. Sublethal exposure to coumaphos reduces the abundance of some bacterial species belonging to the honeybee microbiota, such as *Bifidobacterium* spp. and *Lactobacillus* spp. [21]. Cho et al. [59], in their research, showed the resistance of LAB to the presence of coumaphos and utilization of this pesticide by *L. mesenteroides* WCP907, *L. brevis* WCP902, *L. plantarum* WCP931, and *Latilactobacillus sakei* WCP904 when it was provided as a sole source of phosphorus and carbon [59]. 

In the study above, *P. acidilactici* 8/1 proved to be the most resistant to imidacloprid, with bacterial growth reaching 101.84% ± 1.7 for the insecticide concentration of 100 µg/mL (Figure 1C). According to Cycoń and Piotrowska-Seget [60], imidacloprid negatively affects enzymatic activities and the number of bacteria. Furthermore, imidacloprid increases the growth time and reduces the colony formation rate [60]. Phospholipid fatty acid (PFLA) profiles have shown that imidacloprid significantly reduces biomass and changes the pattern of the microbial community [61]. In the above study, the lack of a negative effect on bacterial growth may be related to LAB resistance to toxic intermediate metabolites resulting from the biodegradation of insecticides. Some bacteria use pesticides as a source of nutrients and energy to multiply. The research carried out by Wang et al. [62] showed that the organophosphate pesticide methamidophos enhances the biomass of Gram-negative bacteria and that it has no significant effect on the growth of Gram-positive bacteria [62]. Some insecticides such as pirimiphos-methyl can inhibit LAB growth but do not affect lactic acid fermentation [63]. The microbiome plays a key role in the health of honeybees. In vitro tests carried out by Jia et al. [64] showed that sublethal doses of imidacloprid do not affect the bacterial diversity in the gut of honeybees. Similar conclusions were noted by Raymann et al. [65], in whose study honeybee gut bacteria were able to grow in the presence of imidacloprid in vitro. The lack of a significant impact of imidacloprid on the bacterial community inhabiting the honeybee microbiota supports our results, where all LAB strains showed high resistance to this insecticide. However, in vivo tests carried out by Almasri et al. [66] showed an effect of imidacloprid on the metabolism of honeybees. Exposure to pesticides may disturb the physiological homeostasis of newly elevated honeybees, thereby increasing the mortality of these insects in the hives [66]. Some pesticides affect the functioning of the LAB, e.g., the production of lactic acid. After the determination of total acidity performed by Abdou et al. [67], endrin and lindane pesticides in concentrations above 2 ppm completely inhibited lactic acid production by LAB. Long-term exposure to imidacloprid leads to intestinal dysbiosis and a reduction in microbial diversity in honeybees [68]. Disturbance of lactic acid production may affect the antimicrobial properties of LAB found in honeybee microbiota, increasing the risk of diseases caused by pathogenic microorganisms [69]. Furthermore, pesticides have an adverse effect on the spore germination of microorganisms [70]. Berber et al. [71] suggested that the disappearance of larvicidal activity in bacteria may be associated with unstable toxic proteins degraded by H+ ion formation and hydroxyl radicals. In research conducted by Abou Ayana et al. [72], pesticides inhibited the growth of all tested strains, and the insecticides Reldan and Lannate contributed to the highest inhibition of bacterial growth [72]. The ABT culture showed the strongest resistance, where the count of LAB reached 11.1 × 10^7^ for the concentration of 7 ppm of Lannate [72].

Strong bacterial growth despite the presence of insecticides is a desirable property for LAB strains showing detoxification potential. Pesticide resistance may be significant when selecting LAB to improve the condition of the microbiota of honeybees that come into direct contact with various types of these chemical substances.

### 3.2. Binding of Pesticides to the Cell Wall of LAB

A binding assay was performed to measure the interactions between LAB and insecticides. In the above study, we determined the ability of 25 LAB strains to bind chlorpyrifos, coumaphos, and imidacloprid (Table 1). Only the concentration of 100 µg/mL of each pesticide was selected for further research, as it is the most commonly used in this type of study [51,52,53,54,55]. All LAB strains absorbed insecticides at various levels. The origin of the LAB did not affect the binding ability. *A kunkeei* DSM 12361, a strain naturally inhabiting the digestive tract of honeybees, showed moderate absorption of the tested pesticides. This suggests the need to strengthen the honeybee microbiota by the application of other probiotic LAB to increase the resistance of these insects to chemical compounds. The tested LAB strains demonstrated a significant ability to bind chlorpyrifos and coumaphos, suggesting the selective activity of these bacteria (*p* ≤ 0.05). The strongest chlorpyrifos absorption was demonstrated by *L. casei* 12AN, *P. parvulus* OK-S, *P. acidilactici* 5/2, *P. pentosaceus* 14/1, and *L. plantarum* 18/1, where the remaining insecticide concentrations were 47.98 ± 0.23, 36.11 ± 0.25, 38.26 ± 0.20, 36.07 ± 0.24, and 58.65 ± 0.28 µg/mL, respectively (Table 1). So, we observed chlorpyrifos binding of up to approx. 64%. In studies carried out by Wang et al. [53], LAB reduced the concentrations of chlorpyrifos contained in rice straw. *L. casei* WYS3 was able to bind 33.3–42% of exogenously added insecticide. Furthermore, the gas chromatography–mass spectrometry analysis suggested detoxification of chlorpyrifos via propargyloxycarbonyl (P-O-C) cleavage [53]. The results in the above study were supported by Trinder et al. [52], in whose study *L. rhamnosus* GG also demonstrated the ability to bind chlorpyrifos. However, the tested LAB strains did not metabolize this insecticide [52].

According to the authors’ knowledge, the absorption of coumaphos by LAB has not been thoroughly investigated. Due to the negative effect of this insecticide on the viability and health of honeybee colonies [73,74,75], the topic needs to be discussed in more detail. In our study, the strongest coumaphos absorption was demonstrated by *L. casei* 12AN, *P. acidilactici* 7/1, *P. pentosaceus* 14/1, *L. plantarum* 14/3, and *P. acidilactici* 35/1, where the remaining insecticide concentrations were 58.95 ± 0.20, 49.38 ± 0.30, 42.96 ± 0.31, 46.28 ± 0.22, and 50.81 ± 0.32 µg/mL, respectively (Table 1). Of all the strains tested, only *P. pentosaceus* 11/3 was not able to bind this pesticide. So, the binding efficiency of coumaphos was up to 57%.

LAB strains exhibited the weakest ability to bind imidacloprid among the tested pesticides, and the remaining insecticide concentration ranged from 63.78 ± 0.45 to 99.10 ± 0.42 µg/mL (Table 1). Only three strains demonstrated higher imidacloprid absorption ability, and these were collection strains *L. plantarum* 8AN, *L. salivarius* 9AN, and *P. parvulus* OK-S. Imidacloprid was bound at the lowest rate, to about 36%. The presented results are similar to those presented in studies by Daisley et al. [55]. All tested LAB strains (*L. rhamnosus* GG, ATCC 27773 and ATCC 7469; *Limosilactobacillus fermentum* 11739 and ATCC 23271; *Limosilactobacillus reuteri* RC14 and *L. plantarum* ATCC 10012) failed to bind imidacloprid during 24 h of co-incubation [55].

Probiotic bacteria can degrade pesticides by producing enzymes and binding to the xenobiotic compounds [76]. The insecticide absorption ability of LAB was tested by Zhang et al. [76]. The results of the assay showed that, after 15 min, *L. plantarum* RS60 and *P. acidilactici* D15 absorbed 55.1% and 56.5% of cypermethrin, respectively [76]. Rezaei et al. [77] presented significant absorption of diazinon by all tested LAB strains. The percentage reduction of this insecticide was within the value range of 56.4–59.8%, where *L. acidophilus* A-9-1 exhibited the strongest binding ability [77]. Recent studies demonstrated the selectivity of LAB in detoxification and pesticide absorption [58,78,79]. According to Yuan et al. [79], phosphate-binding proteins of *L. plantarum* CICC20261 showed various binding energies of organophosphorus pesticides.

In the next step of our research, we demonstrated that some of the tested pesticides were bound by ICEs and MEs of the LAB cell wall (Table 2, Table 3 and Table 4). A higher remaining insecticide concentration was detected in ICEs. The highest concentration of chlorpyrifos was recorded for ICEs and MEs from *P. parvulus* OK-S (21.27 ± 0.39 µg/mL and 2.87 ± 0.18 µg/mL, respectively) (Table 2). A high remaining concentration of this pesticide was also detected in ICEs of *P. acidilactici* 5/2 (6.86 ± 0.15 µg/mL), *P. acidilactici* 7/1 (9.50 ± 0.27 µg/mL), and *L. plantarum* 18/1 (7.37 ± 0.25 µg/mL). The concentration of coumaphos detected in ICEs varied between 0.61 µg/mL for strain 14/1 and 2.23 for *P. acidilactici* 5/2 (Table 3). The strongest binding of imidacloprid was demonstrated by ICEs from *L. salivarius* 9AN, where the remaining insecticide concentration was 7.02 ± 0.11 µg/mL (Table 4). The presented results suggest that the bacterial cell wall components play an important role in binding chemical substances. Furthermore, according to Sreekumar et al. [80], altering the structure of the cell wall may reduce the binding ability of LAB [80]. To better understand the absorption of toxins by LAB, particular attention should be paid to the components of the cell wall. 

Some LAB strains can alleviate insecticide poisoning in vivo [81,82]. Islam et al. demonstrated the ability of *L. brevis* to degrade chlorpyrifos isolated from kimchi [81]. It has been suggested that a serine residue (Ser82) is significantly involved in an enzymatic activity that may be used to detoxify certain pesticides [81]. Zhao and Wang [82] showed that various *Lactobacillus* spp. strains accelerate the degradation of organophosphorus pesticides contained in skimmed milk powder. Among them, malathion turned out to be the most labile and susceptible to the influence of LAB [82]. LAB show potential as a biodetoxificant by detoxifying fungal mycotoxins that can be found in honeybee products. Pollen, due to its pH value, optimal water content, and water activity, is an ideal medium for the development of fungi belonging to *Penicillium*, *Aspergillus*, and *Fusarium* genera [83]. Asurmendi et al. demonstrated in their research the strong binding of aflatoxin B_1_ by various LAB strains [84]. The resulting AFB_1_-LAB complexes were evaluated by sequential washing steps and exhibited strong stability [84].

Strains with the strongest ability to reduce their concentration were selected for the study of pesticide binding capacity by thermally inactivated LAB cells. These were: *L. casei* 12AN, *P. parvulus* OK-S, *P. acidilactici* 5/2, *P. pentosaceus* 14/1, and *L. plantarum* 18/1 (chlorpyrifos); *L. casei* 12AN, *P. acidilactici* 7/1, *P. pentosaceus* 14/1, *L. plantarum* 14/3, and *P. acidilactici* 35/1 (coumaphos); and *L. plantarum* 8AN, *L. salivarius* 9AN, and *P. parvulus* OK-S (imidacloprid). However, in the heat-inactivated form (dead cells), these strains did not show any pesticide binding capacity. The concentration of insecticide detected with HPLC was the same as the initial concentration. This confirms the definition of probiotics, i.e., that they must only be live microbial cells. The effect of LAB viability on their pesticide absorption capacity needs to be discussed in more detail. The above-mentioned strains were selected for further cyto- and genotoxicity studies on cell lines.

The above-mentioned LAB strains showing the most potent insecticide binding capacity were additionally tested in order to evaluate their ability to metabolize these compounds. For this, a minimal medium was elaborated, and it was composed of: sodium dihydrogen phosphate dodecahydrate (5.95 g/L), potassium dihydrogen phosphate (2.27 g/L), sodium chloride (1 g/L), magnesium sulfate heptahydrate (0.5 g/L), calcium chloride dihydrate (0.01 g/L), manganese sulfate tetrahydrate (0.02 g/L), ferrous sulfate heptahydrate (0.05 g/L), and peptone from casein (0.01 g/L) [57]. Simultaneously, the same experiment was conducted with the usage of MRS medium. The samples were incubated for 24, 48, 72, and 96 h at 30 °C and then subjected to HPLC analysis. We observed no reduction in the concentration of insecticides in the minimal or MRS medium at any time point (within the limits of SD). Therefore, no metabolites were determined. We concluded that the reduction in the concentration of the tested compounds was not the result of the metabolism but of the binding to the cell wall. 

### 3.3. Detoxification of Pesticides by LAB

In the presented study, the authors tested the reduction in cytotoxicity and genotoxicity of pesticides by LAB. As there is no permanent cell line isolated from *Apis mellifera* L., Sf-9 cells were selected for the study as they are the most established insect cells suitable for certain applications in honeybee research and are used in toxicity studies [85]. Additionally, two more intestinal cell lines were chosen. In culture, Caco-2 cancer intestinal epithelial cells exhibit similar morphological, functional, and structural properties to those of intestinal enterocytes. For this reason, this cell line is the most common in vitro cell-based research model for gastrointestinal and probiotic–LAB interactions. IEC-6 normal intestinal cells are a suitable cell model for in vitro testing as they possess the cosmic enterocyte phenotype occurring in vivo.

For the present experiments, those samples that were analyzed by HPLC were taken (Table 1), and the LAB strains with the highest binding capacity of the tested pesticides were selected.

#### 3.3.1. Decrease in cytotoxicity

**Figure 2:**
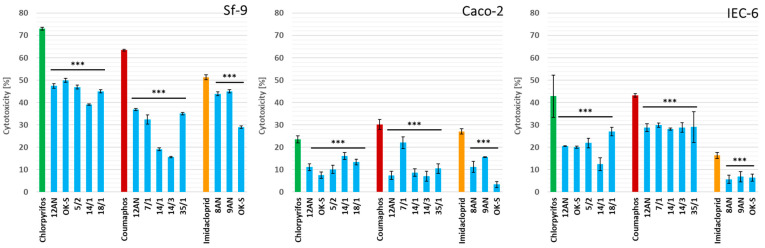
Decrease in cytotoxicity of chlorpyrifos, coumaphos, and imidacloprid after 24 h incubation with lactic acid bacteria (LAB) strains measured by MTT (3-(4,5-dimethylthiazol-2-yl)-2,5-diphenyltetrazolium bromide) assay. Each data point represents the mean of the absorbance values from cells from eight individual wells. Results are presented as mean ± standard deviation (SD). Statistical significance was calculated versus positive control (appropriate pesticide standard), * *p* ≤ 0.1, ** *p* ≤ 0.05, *** *p* ≤ 0.01.

In the above study, we evaluated the effect of LAB strains on the reduction in insecticide cytotoxicity (Figure 2). All tested LAB strains significantly decreased the cytotoxicity of chlorpyrifos, coumaphos, and imidacloprid (*p* ≤ 0.01) in all tested cell lines. 

The greatest cytotoxicity of tested insecticides among the cell lines was noted for Sf-9 cells. For these cells, LAB strains showed the highest degree of detoxification of coumaphos, and the cytotoxicity ranged from 15.53% ± 0.32 for *L. plantarum* 14/3 to 36.92% ± 0.48 for *L. casei* 12AN, and this was significantly different from the results obtained for the pure pesticide (*p* ≤ 0.01). So, the highest degree of coumaphos detoxification (75.51%) was demonstrated by *L. plantarum* 14/3. The results obtained for chlorpyrifos and imidacloprid also showed significant decreases in cytotoxicity, and the degrees of their detoxification ranged from 31.69% for *P. parvulus* OK-S to 46.50% for *P. pentosaceus* 14/1 and from 12.29% for *L. salivarius* 9AN to 43.54% for *P. parvulus* OK-S, respectively (*p* ≤ 0.01). The strong detoxification of insecticides in the Sf-9 cell line sheds new light on the role of LAB in protecting honeybees from various chemical substances. Strengthening the microbiota of honeybees with selected LAB strains may result in the improvement of their resistance to the negative effects of pesticides. 

The results obtained for the cancerous cell line Caco-2 showed a particularly large decrease in the cytotoxicity of imidacloprid due to the potentially probiotic properties of the tested LAB strains (Figure 2). The degree of detoxification of this insecticide reached 87.60% for *P. parvulus* OK-S, which almost completely reduced its cytotoxicity. A particularly high degree of detoxification of coumaphos was demonstrated by *P. pentosaceus* 14/1, significantly reducing its cytotoxicity (*p* ≤ 0.01). The degree of detoxification of chlorpyrifos reached 67.99% for *P. parvulus* OK-S (*p* ≤ 0.01).

In the above study, the results obtained for IEC-6 cell line showed the most potent reduction in the cytotoxicity of chlorpyrifos and imidacloprid (Figure 2). The degree of detoxification of chlorpyrifos reached 70.91% for *P. pentosaceus* 14/1. The remaining LAB strains also reduced the cytotoxicity of this insecticide, demonstrating the strong influence of LAB on its toxic effects. The cytotoxicity of imidacloprid was decreased to 5.68% for *L. plantarum* 8AN and 6.40% for *P. parvulus* OK-S, which is significantly different from the result obtained for the positive control (*p* ≤ 0.01). The highest degree of detoxification, 65.19%, was demonstrated by *L. plantarum* 8AN (*p* ≤ 0.01). 

Various chemical substances have a toxic effect on living cells [86]. In vitro tests on the Caco-2 cell line showed the cytotoxic effect of deltamethrin, fenitrothion, fipronil, lambda-cyhalothrin, and teflubenzuron [87]. Fipronil and fenitrothion significantly influenced lipid peroxidation and the activity of antioxidant enzymes and disturbed the integrity of the cell layer. The presence of antioxidants reduced lipid peroxidation levels, suggesting that the key mechanism of pesticide cytotoxicity is related to their pro-oxidative potential [87]. Abhishek et al. [88], in their research, determined the cytotoxic effect of alpha-hexachlorocyclohexane, parathion methyl, and carbofuran on the human keratinocyte (HaCaT) cell line [88]. The greatest cytotoxicity was noted for pesticide mixtures, suggesting a synergistic mechanism in the action of these xenobiotics [88]. According to the results of the MTT assay performed by Abdel-Halim and Osman, imidacloprid induces oxidative stress and apoptotic effects in the prostate epithelial WPM-Y1 cell line [89]. The median inhibition values for 24 h were 0.023 mM, and the presence of the pesticide significantly changed the levels of cellular enzymes [89]. Some insecticides have a cytotoxic effect on insect cells, inhibit cell viability, and affect cell proliferation [90]. It has also been suggested that cytotoxicity varies with cell type, pesticide, and concentration [90]. The study carried out by Pandya et al. [91] suggested that Profex and Ammo (broad-spectrum insecticides) exhibit significant cytotoxicity to the insect Sf-9 cell line. Acridine ethidium bromide staining confirmed the toxic potential of the pesticides and demonstrated apoptotic cell death upon exposure [91]. According to the authors’ knowledge, studies on the influence of LAB on the cytotoxicity of pesticides are very limited. In order to better understand the probiotic properties of LAB, the reduction in the cytotoxicity of chemical substances shown by these bacteria should be further evolved.

The reduction in cytotoxicity of the tested pesticides was correlated with a decrease in their concentration in the cell wall binding assay. More sensitive than cancerous cells (Caco-2) to the tested compounds were normal cells (Sf-9 and IEC-6). The detoxification depended on insecticide and cell line and was a specific feature of an individual LAB strain. Comparing all the results presented in the study above for the reduction of pesticide cytotoxicity, the authors suggest a strong influence of LAB on the negative effects caused by chemical substances. For all three cell lines, the tested LAB strains significantly influenced the cytotoxicity of the pesticides. Therefore, there is a high probability that some LAB strains are able to protect the intestinal cells of honeybees, thereby increasing the viability of these insects.

#### 3.3.2. Decrease in Genotoxicity

**Figure 3:**
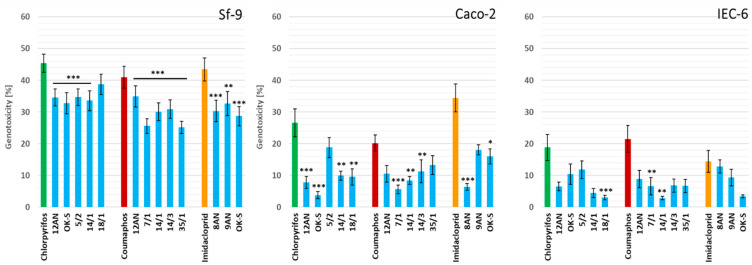
Decrease in the genotoxicity of chlorpyrifos, coumaphos, and imidacloprid after 24 h incubation with lactic acid bacteria (LAB) strains expressed as the mean percentage of DNA in the comet tail in the alkaline comet assay. Fifty cells were analyzed for each treatment. Results are presented as mean ± standard error of the mean (S.E.M.). Statistical significance was calculated versus positive control (appropriate pesticide standard), * *p* ≤ 0.1, ** *p* ≤ 0.05, *** *p* ≤ 0.01.

In the study, we assessed the effect of LAB strains on the reduction in genotoxicity of tested pesticides (Figure 3). All tested LAB strains showed a reduction in the genotoxic effects of pesticides in all cell lines. 

Sf-9 cells, again, showed the highest sensitivity to the tested insecticides (Figure 3). LAB strains displayed a significant reduction in the genotoxicity of imidacloprid (*p* ≤ 0.05). The mean genotoxicity of imidacloprid after incubation with LAB strains ranged from 28.68% ± 3.0 for *P. parvulus* OK-S to 32.62% ± 3.80 for *L. salivarius* 9AN, and the highest detoxification degree (approx. 34%) was demonstrated by *P. parvulus* OK-S. LAB strains also reduced the genotoxicity of chlorpyrifos and coumaphos. The degree of detoxification of these insecticides ranged from 14.69% for *L. plantarum* 18/1 to 27.79% for *P. parvulus* OK-S and from 14.77% for *L. casei* 12AN to 38.65% for *P. acidilactici* 35/1, respectively. The mean genotoxicity was significantly different from the results obtained for the control sample (pure insecticide), suggesting the influence of LAB on the genotoxic effects of pesticides (*p* ≤ 0.05). 

Caco-2 cells showed the greatest sensitivity to imidacloprid and chlorpyrifos (Figure 3). All tested LAB strains reduced the genotoxicity of insecticides in this cell line. Detoxification of chlorpyrifos reached 85.84% for *P. parvulus* OK-S. The mean imidacloprid genotoxicity was the lowest after incubation with *L. plantarum* 8AN, showing a detoxification degree of 81.52%. All LAB strains decreased the genotoxicity of coumaphos, suggesting their strong ability to detoxify this pesticide. The degree of detoxification of this insecticide ranged from 34.33% for *P. acidilactici* 35/1 to 72.25% for *P. acidilactici* 7/1.

**Figure 4:**
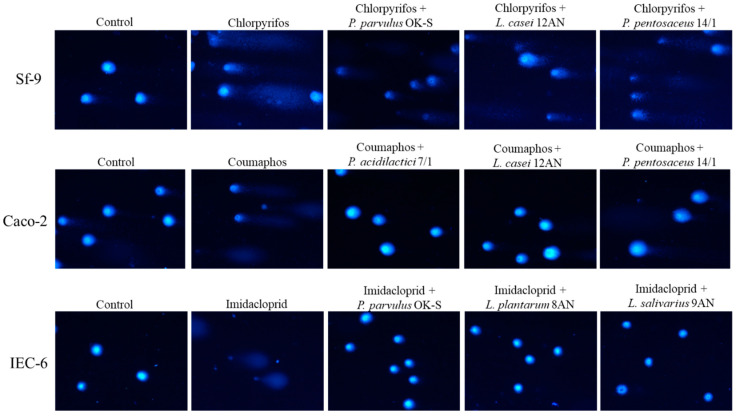
Representative images of comets stained with 1 µ/mL DAPI, fluorescence microscopy (Nikon, Tokyo, Japan), 20× objective.

The results obtained for pesticide detoxification in the IEC-6 cell line demonstrated a particularly potent reduction in the genotoxicity of coumaphos (Figure 3). The highest degree of detoxification of this insecticide, equal to 86.89%, was demonstrated by *P. pentosaceus* 14/1 (*p* ≤ 0.05). The mean genotoxicity of coumaphos after incubation with LAB strains ranged from 2.82% ± 0.52 for *P. pentosaceus* 14/1 to 8.84% ± 2.74 for *L. casei* 12AN. The second most strongly detoxified pesticide by LAB strains was chlorpyrifos. The highest degree of detoxification of this xenobiotic (83.87%) was demonstrated by *L. plantarum* 18/1 (*p* ≤ 0.01). LAB strains showed varying levels of reduction in the genotoxicity of imidacloprid. The degree of detoxification of this pesticide ranged from 11.01% for *L. plantarum* 8AN to 76.32% for *P. parvulus* OK-S, but it was not statistically significant. Example comet images are shown in Figure 4.

The obtained results typically showed a strong reduction in insecticide genotoxicity in all three cell lines. The ability depended on the cell line, insecticide, and LAB strain and was rather strain specific. Reducing the genotoxicity of pesticides in cells found in the digestive tract of honeybees is a key step in increasing the resistance of these insects to toxins from the external environment. Genotoxicity can be defined as genetic alterations that are induced by genotoxins, such as structural chromosomal aberrations, gene mutations, and DNA recombination [92]. Various pesticides demonstrate the ability to damage the genetic information of the cell [93]. According to Kizılet et al. [94], dimethoate shows genotoxic effects on human peripheral lymphocytes and introduces genetic alterations even at low concentrations [94]. The degree of genotoxicity of some pesticides depends on the doses and the time of exposure [95]. Genotoxic assays performed by Khodabandeh et al. [96] on extracted cells from bone marrow showed DNA degradation after 5 days of exposure to zolone. The genotoxicity of some insecticides suggests that we should have serious concerns about their potential effects on living organisms [97]. According to Chandrakar et al. [97], malathion and carbofuran alter chromosomal aberrations, comet tail length, micronucleus formation, and comet scores of cat (*Felis catus*) fibroblast cells. Malathion promoted genomic instability at high concentrations, and maximum DNA damage was recorded at 45 mM of pesticide [97]. Some organochlorine pesticides have a genotoxic effect on buccal cells, influencing the abnormality of the cell nucleus and apoptosis [98]. Using a comet assay, Saleh et al. [99] determined the genotoxicity of lambda-cyhalothrin on the insect Sf-9 cell line. Most of the insecticide concentrations showed significant increases in DNA damage in cells. Moreover, lambda-cyhalothrin induced oxidative stress in cell lines, enhancing the cytotoxic effect caused by this pesticide [99]. According to the authors’ knowledge, there are no in-depth studies on the reduction of pesticide genotoxicity by LAB. Honeybees are exposed to toxic chemicals that can genetically alter cells and contaminate honey and honeybee products (such as propolis or bee pollen) also eaten by humans [100,101,102]. Contamination of honeybee products can occur through the transport of toxic chemicals by honeybees to the hives, having been indirectly collected from environmental sources via water, air, and soil and directly from agricultural practices [103]. The concentration of chlorpyrifos in honey depends on the origin of the sample [103]. According to studies conducted in Uruguay, chlorpyrifos is the most detectable compound in honey, with a highest concentration of 80 ng/g [104]. Similar results were obtained after testing honey from various agricultural areas in Greece, with chlorpyrifos concentrations ranging from 0.70 to 0.89 ng/g [105]. Despite the European moratorium, low concentrations of imidacloprid (<2 ng/g) are still detectable in honey. Some plants, with which honeybees are in constant contact, grow in recently contaminated soil, which may explain the residual imidacloprid in pollen and nectar [106]. In studies carried out by Valdovinos-Flores et al., coumaphos was detected in 64% of honey samples and 100% of wax samples collected from Yucatan [107]. The concentration of this pesticide reached 0.04 mg/kg and exceeded the maximum residue limit allowed in some countries [107]. Humans are at risk of exposure to pesticides through the heavy use of these chemicals in agriculture. According to Tao et al., the level of exposure to imidacloprid depends on age and place of residence [108]. People living in urban areas are less likely to be exposed to the negative effects of insecticides [108]. In a study conducted on 68 patients from three hospitals in Sri Lanka, the median amount of ingested imidacloprid was 15 mL. Most of the patients presented with symptoms such as headache, diarrhea, nausea, and vomiting [109]. The World Health Organization (WHO) classifies organophosphate pesticides as either extremely toxic or moderately toxic to human health [110]. According to Taheri et al., metabolites of chlorpyrifos were detected in over 70% of samples, with an average concentration of 3.80 ± 2.72 µg/L [110]. 

Supporting the health of honeybees through the application of selective probiotic LAB would also protect humans, i.e., consumers of honeybee products, from the negative effects of pesticides. LAB with detoxifying properties could be administered to honeybees prophylactically with food, reducing the possibility of human consumption of contaminated honeybee products. In the above study, LAB strains showed the potent ability to detoxify insecticides, which, therefore, provides additional support for the honeybee microbiota. Our study had several strengths. A wide variety of in vitro tests can be conducted with various options on a large number of LAB strains by screening. The results of the presented study offer favorable prospects for the detoxification of various toxic substances that threaten the health of animals and humans. LAB can contribute to research into the reduction of cytotoxicity and genotoxicity of a wider spectrum of chemicals with negative effects on various cell lines. This study also had some limitations. The laboratory conditions were artificial, and, therefore, in vivo testing must be performed, especially since the honeybees’ digestive tract is also inhabited by microbiota other than LAB, and it is not known how they might interact with pesticides. 

## 4. Conclusions

Honeybees are constantly exposed to various harmful chemicals, especially pesticides. Exposure to these compounds can have negative effects on the health of honeybees, and there is a growing need to increase the resistance of these pollinators. LAB exhibit a broad spectrum of detoxification of different chemicals while being safe microorganisms classified as probiotics. In the above study, all LAB strains showed high resistance to chlorpyrifos, coumaphos, and imidacloprid. The potent growth of bacteria, despite the presence of pesticides, is a desirable feature for microorganisms that are supposed to perform probiotic and detoxification functions. It was observed that LAB resistance to coumaphos and chlorpyrifos was contingent on the origin of the strain; however, it did not affect their binding ability by bacteria. All tested LAB strains showed various levels of insecticide binding and more potent capacity compared to *A. kunkeei* DSM 12361, a strain that naturally inhabits the gut of honeybees. Heat-inactivated LAB strains did not show the ability to bind pesticides; however, the impact of the viability of these bacteria on their detoxification capacity needs to be studied in greater detail. The results suggest that the detoxification of insecticides is a strain-dependent feature and also depends on the cell line on which the experiment was performed and probably is connected with the chemical structure of the insecticide. The insecticide toxicity reduction was unique to each LAB strain and varied with the chemical tested. *P. pentosaceus* 14/1 exhibited the highest degree of detoxification in Sf-9 cell assays while showing the most potent ability to bind chlorpyrifos and coumaphos. The LAB strains that also demonstrated a significant reduction in toxicity of the tested insecticides were: *P. parvulus* OK-S, *L. plantarum* 18/1, *L. salivarius* 9AN, and *L. casei* 12AN. Due to the role of the honeybee microbiota, it is important to find a natural way to support and protect it from toxic substances. Disruption of the microbial structure in the honeybee gut may result in a reduction in the resistance of these pollinators to diseases caused by honeybee pathogens and, consequently, lead to an increase in mortality in honeybee colonies. The detoxification of pesticides in honeybees could reduce the likelihood of the penetration of toxins into the human body along with consumed honeybee products and honey. The LAB strains that exhibited the most potent detoxification abilities against the tested pesticides will be selected for future in vitro tests, such as tests relating to antibiotic resistance, biofilm formation, survival in sugar syrup, or in simulated gastrointestinal conditions. The conducted research requires confirmation in vivo and suggests the great potential of LAB in the construction of a health-promoting preparation for honeybees which increases the resistance of these insects against various pesticides. 

## Figures and Tables

**Table 1:** The ability of lactic acid bacteria (LAB) strains to bind pesticides to the cell wall. The experiments were conducted for whole live LAB cells. The initial concentration of an individual pesticide was 100 µg/mL. The presented results are mean from two measurements ± standard deviation (SD). Statistical significance was calculated versus positive control (appropriate pesticide standard, 100 µg/mL), * *p* ≤ 0.05.

Strain	Source	Remaining Pesticide Concentration (µg/mL) ± SD		
		Chlorpyrifos	Coumaphos	Imidacloprid
*A. kunkeei* DSM 12361	Honeybee gut	94.44 ± 0.34	70.29 ± 0.30 *	97.70 ± 0.25
*L. rhamnosus* GG	Human feces	72.26 ± 0.45 *	87.28 ± 0.41 *	94.83 ± 0.31
*L. plantarum* 8AN	Chicken feces	85.68 ± 0.31 *	83.60 ± 0.38 *	81.88 ± 0.55 *
*L. salivarius* 9AN	Chicken feces	94.21 ± 0.45	67.37 ± 0.31 *	63.78 ± 0.45 *
*L. casei* 12AN	Human feces	47.98 ± 0.23 *	58.95 ± 0.20 *	90.74 ± 0.25
*P. parvulus* OK-S	Fermented cucumbers	36.11 ± 0.25 *	93.30 ± 0.29	73.12 ± 0.21 *
*L. brevis* KKA	Fermented cabbage	66.98 ± 0.44 *	77.85 ± 0.38 *	99.10 ± 0.42
*L. plantarum* 145	Vegetable silage	65.46 ± 0.38 *	75.05 ± 0.20 *	95.91 ± 0.45
*L. acidophilus* 573	Nd ^a^	100.31 ± 0.48	77.97 ± 0.51 *	87.30 ± 0.61 *
*P. acidilactici* 4/1	*Robinia pseudoaccacia* L. ^b^	74.75 ± 0.40 *	92.15 ± 0.35	90.96 ± 0.31
*P. acidilactici* 5/2	*Weigela florida* DC. ^b^	38.26 ± 0.20 *	60.28 ± 0.25 *	97.54 ± 0.21
*P. acidilactici* 6/1	*Centaurea jacea* L. ^b^	84.88 ± 0.45 *	60.70 ± 0.50 *	84.22 ± 0.48 *
*P. acidilactici* 7/1	*Papaver rhoeas* L. ^b^	62.04 ± 0.30 *	49.38 ± 0.30 *	95.54 ± 0.33
*P. acidilactici* 8/1	*Sinapis arvensis* L. ^b^	82.36 ± 0.41 *	61.37 ± 0.35 *	94.19 ± 0.27
*P. pentosaceus* 9/3	*Trifolium pratense* L. ^b^	99.08 ± 0.50	68.23 ± 0.40 *	98.47 ± 0.42
*P. pentosaceus* 10/2	*Sambucus nigra* L. ^b^	69.18 ± 0.33 *	97.84 ± 0.50	92.24 ± 0.40
*P. pentosaceus* 11/3	*Philadelphus coronaries* L. ^b^	64.91 ± 0.30 *	100.97 ± 0.89	97.23 ± 0.29
*P. pentosaceus* 14/1	*Lavandula augustifolia* L. ^b^	36.07 ± 0.24 *	42.96 ± 0.31 *	98.32 ± 0.29
*L. plantarum* 14/3	*Lavandula augustifolia* L. ^b^	61.84 ± 0.30 *	46.28 ± 0.22 *	97.91 ± 0.51
*L. plantarum* 18/1	*Buddleja davidii* L. ^b^	58.65 ± 0.28 *	82.96 ± 0.44 *	99.08 ± 0.18
*P. pentosaceus* 19/1	*Calluna vulgaris* L. ^b^	98.99 ± 0.47	69.20 ± 0.30 *	93.40 ± 0.39
*L. plantarum* 21/1	Fermented (spoiled) honey	61.56 ± 0.29 *	94.08 ± 0.29	97.45 ± 0.38
*P. acidilactici* 22/1	Royal jelly	92.94 ± 0.46	91.83 ± 0.67	91.13 ± 0.68
*P. acidilactici* 25/1	Heather nectar honey	74.18 ± 0.31 *	89.23 ± 0.81 *	98.42 ± 0.40
*P. acidilactici* 35/1	Goldenrod honey	96.18 ± 0.48	50.81 ± 0.32 *	84.22 ± 0.36 *
Analytical standard	-	100.00 ± 0.86	100.00 ± 0.99	100.00 ± 0.29

^a^ Nd—no data; ^b^ flowers.

**Table 2:** Amount of chlorpyrifos bound to intracellular extracts and membrane extracts after 24 h incubation with selected strains of lactic acid bacteria (LAB). The experiments were conducted for whole live LAB cells. The initial concentration of an individual pesticide was 100 µg/mL. The presented results are mean from two measurements ± SD.

Strain	Remaining Chlorpyrifos Concentration (µg/mL) ± SD	
	Intracellular Extracts	Membrane Extracts
*L. casei* 12AN	0.31 ± 0.07	0.03 ± 0.01
*P. parvulus* OK-S	21.27 ± 0.39	2.87 ± 0.18
*L. brevis* KKA	2.49 ± 0.13	0.28 ± 0.02
*L. plantarum* 145	1.42 ± 0.10	0.19 ± 0.05
*P. acidilactici* 5/2	6.86 ± 0.15	0.72 ± 0.07
*P. acidilactici* 7/1	9.50 ± 0.27	2.09 ± 0.10
*P. pentosaceus* 10/2	0.27 ± 0.03	0.11 ± 0.03
*P. pentosaceus* 11/3	0.74 ± 0.09	0.11 ± 0.04
*P. pentosaceus* 14/1	2.70 ± 0.17	0.19 ± 0.01
*L. plantarum* 14/3	2.02 ± 0.15	0.41 ± 0.04
*L. plantarum* 18/1	7.37 ± 0.25	0.62 ± 0.10
*L. plantarum* 21/1	1.10 ± 0.12	0.10 ± 0.03
Analytical standard	100.00 ± 0.45	

**Table 3:** Amount of coumaphos bound to intracellular extracts and membrane extracts after 24 h incubation with selected strains of lactic acid bacteria (LAB). The experiments were conducted for whole live LAB cells. The initial concentration of an individual pesticide was 100 µg/mL. The presented results are mean from two measurements ± SD.

Strain	Remaining Coumaphos Concentration (µg/mL) ± SD	
	Intracellular Extracts	Membrane Extracts
*L. salivarius* 9AN	1.45 ± 0.07	0.11 ± 0.05
*L. casei* 12AN	1.81 ± 0.10	0.13 ± 0.05
*P. acidilactici* 5/2	2.23 ± 0.13	0.25 ± 0.07
*P. acidilactici* 6/1	1.53 ± 0.12	0.13 ± 0.03
*P. acidilactici* 7/1	1.71 ± 0.10	0.09 ± 0.02
*P. acidilactici* 8/1	1.13 ± 0.08	0.17 ± 0.05
*P. pentosaceus* 9/3	1.40 ± 0.06	0.13 ± 0.03
*P. pentosaceus* 14/1	0.61 ± 0.04	0.12 ± 0.04
*L. plantarum* 14/3	0.79 ± 0.06	0.02 ± 0.01
*P. pentosaceus* 19/1	0.89 ± 0.05	0.04 ± 0.01
*P. acidilactici* 35/1	1.89 ± 0.10	0.19 ± 0.03
Analytical standard	100.00 ± 0.66	

**Table 4:** Amount of imidacloprid bound to intracellular extracts and membrane extracts after 24 h incubation with selected strains of lactic acid bacteria (LAB). The experiments were conducted for whole live LAB cells. The initial concentration of an individual pesticide was 100 µg/mL. The presented results are mean from two measurements ± SD.

Strain	Remaining Imidacloprid Concentration (µg/mL) ± SD	
	Intracellular Extracts	Membrane Extracts
*L. plantarum* 8AN	2.44 ± 0.13	0.60 ± 0.08
*L. salivarius* 9AN	7.02 ± 0.11	1.28 ± 0.10
*P. parvulus* OK-S	5.99 ± 0.19	1.34 ± 0.11
Analytical standard	100.00 ± 0.72	

## Data Availability

The data presented in this study are available in this article and are available from the corresponding author upon reasonable request.

## References

[1] Abhilash P.C., Singh N. (2009). Pesticide use and application: An Indian scenario. J. Hazard. Mater..

[2] Fries G.F., Marrow G.S., Gordon C.H. (1969). Comparative excretion and retention of DDT analogs by Dairy Cows. J. Dairy Sci..

[3] Osman K.A. (2011). Pesticides and human health. Pesticides in the Modern World—Effects of Pesticides Exposure.

[4] Aktar W., Sengupta D., Chowdhury A. (2009). Impact of pesticides use in agriculture: Their benefits and Hazards. Interdiscip. Toxicol..

[5] Fenner-Crisp P.A. (2010). Risk assessment and risk management. Hayes’ Handbook of Pesticide Toxicology.

[6] Feazel-Orr H.K., Catalfamo K.M., Brewster C.C., Fell R.D., Anderson T.D., Traver B.E. (2016). Effects of Pesticide Treatments on Nutrient Levels in Worker Honey Bees (*Apis mellifera*). Insects.

[7] Wu J.Y., Anelli C.M., Sheppard W.S. (2011). Sub-lethal effects of pesticide residues in brood comb on worker honey bee (*Apis mellifera*) development and longevity. PLoS ONE.

[8] Wahl O., Ulm K. (1983). Influence of pollen feeding and physiological condition on pesticide sensitivity of the honey bee *Apis mellifera carnica*. Oecologia.

[9] Claudianos C., Ranson H., Johnson R.M., Biswas S., Schuler M.A., Berenbaum M.R., Feyereisen R., Oakeshott J.G. (2006). A deficit of detoxification enzymes: Pesticide sensitivity and environmental response in the honeybee. Insect Mol. Biol..

[10] Kumar G., Singh S., Pramod Kodigenahalli Nagarajaiah R. (2020). Detailed review on pesticidal toxicity to honey bees and its management. Modern Beekeeping—Bases for Sustainable Production.

[11] Martelli F., Zhongyuan Z., Wang J., Wong C.-O., Karagas N.E., Roessner U., Rupasinghe T., Venkatachalam K., Perry T., Bellen H.J. (2020). Low doses of the neonicotinoid insecticide imidacloprid induce ROS triggering neurological and metabolic impairments in *Drosophila*. Proc. Natl. Acad. Sci. USA.

[12] Tan J., Galligan J.J., Hollingworth R.M. (2007). Agonist actions of neonicotinoids on nicotinic acetylcholine receptors expressed by cockroach neurons. NeuroToxicology.

[13] Tosi S., Nieh J.C., Sgolastra F., Cabbri R., Medrzycki P. (2017). Neonicotinoid pesticides and nutritional stress synergistically reduce survival in honey bees. Proc. R. Soc. B..

[14] Laycock I., Lenthall K.M., Barratt A.T., Cresswell J.E. (2012). Effects of imidacloprid, a neonicotinoid pesticide, on reproduction in worker bumble bees (*Bombus terrestris*). Ecotoxicology.

[15] Urlacher E., Monchanin C., Rivière C., Richard F.-J., Lombardi C., Michelsen-Heath S., Hageman K., Mercer A. (2016). Measurements of chlorpyrifos levels in forager bees and comparison with levels that disrupt honey bee odor-mediated learning under laboratory conditions. J. Chem. Ecol..

[16] Cutler G.C., Purdy J., Giesy J.P., Solomon K.R. (2014). Risk to pollinators from the use of chlorpyrifos in the United States. Ecological Risk Assessment for Chlorpyrifos in Terrestrial and Aquatic Systems in the United States.

[17] Darko G., Addai Tabi J., Adjaloo M.K., Borquaye L.S. (2017). Pesticide residues in honey from the major honey producing forest belts in Ghana. J. Environ. Public Health.

[18] Hites R.A. (2021). The rise and fall of chlorpyrifos in the United States. Environ. Sci. Technol..

[19] Ola-Davies O.E., Azeez O.I., Oyagbemi A.A., Abatan M.O. (2018). Acute coumaphos organophosphate exposure in the domestic dogs: Its implication on haematology and liver functions. Int. J. Vet. Sci. Med..

[20] Berry J.A., Hood W.M., Pietravalle S., Delaplane K.S. (2013). Field-level sublethal effects of approved bee hive chemicals on honey bees (*Apis mellifera* L). PLoS ONE.

[21] Rouzé R., Moné A., Delbac F., Belzunces L., Blot N. (2019). The honeybee gut microbiota is altered after chronic exposure to different families of insecticides and infection by *Nosema ceranae*. Microbes Environ..

[22] Wang K., Chen H., Fan R.-L., Lin Z.-G., Niu Q.-S., Wang Z., Ji T. (2022). Effect of Carbendazim on Honey Bee Health: Assessment of survival, pollen consumption, and gut microbiome composition. Ecotoxicol. Environ. Saf..

[23] Balbuena S., Castelli L., Zunino P., Antúnez K. (2022). Effect of chronic exposure to sublethal doses of imidacloprid and *Nosema ceranae* on immunity, gut microbiota, and survival of Africanized honey bees. Microb. Ecol..

[24] Kakumanu M.L., Reeves A.M., Anderson T.D., Rodrigues R.R., Williams M.A. (2016). Honey bee gut microbiome is altered by in-hive pesticide exposures. Front. Microbiol..

[25] Motta E.V., Raymann K., Moran N.A. (2018). Glyphosate perturbs the gut microbiota of Honey Bees. Proc. Natl. Acad. Sci. USA.

[26] Cuesta-Maté A., Renelies-Hamilton J., Kryger P., Jensen A.B., Sinotte V.M., Poulsen M. (2021). Resistance and vulnerability of honeybee (*Apis mellifera*) gut bacteria to commonly used pesticides. Front. Microbiol..

[27] Mathialagan M., Thangaraj Edward Y.S.J., David P.M.M., Senthilkumar M., Srinivasan M.R., Mohankumar S. (2018). Isolation, characterization and identification of probiotic lactic acid bacteria (LAB) from honey bees. Int. J. Curr. Microbiol. Appl. Sci..

[28] Nowak A., Szczuka D., Górczyńska A., Motyl I., Kręgiel D. (2021). Characterization of *Apis mellifera* Gastrointestinal Microbiota and Lactic Acid Bacteria for Honeybee Protection—A Review. Cells.

[29] Pop O.L., Suharoschi R., Gabbianelli R. (2022). Biodetoxification and protective properties of probiotics. Microorganisms.

[30] Petrova P., Arsov A., Tsvetanova F., Parvanova-Mancheva T., Vasileva E., Tsigoriyna L., Petrov K. (2022). The complex role of lactic acid bacteria in food detoxification. Nutrients.

[31] Alcántara C., Jadán-Piedra C., Vélez D., Devesa V., Zúñiga M., Monedero V. (2017). Characterization of the binding capacity of mercurial species in *Lactobacillus* strains. J. Sci. Food Agric..

[32] Sadiq F.A., Yan B., Tian F., Zhao J., Zhang H., Chen W. (2019). Lactic acid bacteria as antifungal and anti-mycotoxigenic agents: A comprehensive review. Compr. Rev. Food. Sci. Food Saf..

[33] Nowak A., Libudzisz Z. (2009). Ability of probiotic *Lactobacillus casei* DN 114001 to bind or/and metabolise heterocyclic aromatic amines in vitro. Eur. J. Nutr..

[34] Nowak A., Czyżowska A., Stańczyk M. (2015). Protective activity of probiotic bacteria against2-amino-3-methyl-3H-imidazo[4,5-f]quinoline (IQ) and 2-amino-1-methyl-6-phenyl-1H-imidazo[4,5-b]pyridine (PhIP)—An in vitro study. Food Addit. Contam. A.

[35] Yousefi M., Khorshidian N., Hosseini H. (2021). The ability of probiotic *Lactobacillus* strains in removal of benzo[a]pyrene: A response surface methodology study. Probiotics Antimicrob..

[36] Shoukat S. (2020). Potential anti-carcinogenic effect of probiotic and lactic acid bacteria in detoxification of benzo[a]pyrene: A Review. Trends Food Sci. Technol..

[37] Yousefi M., Shariatifar N., Tajabadi Ebrahimi M., Mortazavian A.M., Mohammadi A., Khorshidian N., Arab M., Hosseini H. (2019). In vitro removal of polycyclic aromatic hydrocarbons by lactic acid bacteria. J. Appl. Microbiol..

[38] Lili Z., Junyan W., Hongfei Z., Baoqing Z., Bolin Z. (2017). Detoxification of cancerogenic compounds by lactic acid bacteria strains. Crit. Rev. Food. Sci. Nutr..

[39] Barbasz A., Kreczmer B., Skórka M., Czyżowska A. (2020). Toxicity of pesticides toward human immune cells U-937 and HL-60. J. Environ. Sci. Health B.

[40] Cestonaro L.V., Macedo S.M., Piton Y.V., Garcia S.C., Arbo M.D. (2022). Toxic effects of pesticides on cellular and humoral immunity: An overview. Immunopharmacol. Immunotoxicol..

[41] Kumar N., Pathera A., Saini P., Kumar M. (2012). Harmful effects of pesticides on human health. Ann. Agric. Bio. Res..

[42] Issaragrisil S., Chansung K., Kaufman D.W., Sirijirachai J., Thamprasit T., Young N.S. (1997). Aplastic anemia in rural Thailand: Its association with grain farming and agricultural pesticide exposure. aplastic anemia study group. Am. J. Public Health.

[43] Naughton S.X., Terry A.V. (2018). Neurotoxicity in acute and repeated organophosphate exposure. Toxicology.

[44] Nasrollahzadeh A., Mokhtari S., Khomeiri M., Saris P. (2022). Mycotoxin detoxification of food by lactic acid bacteria. Int. J. Food Contam..

[45] Chmiel J.A., Daisley B.A., Pitek A.P., Thompson G.J., Reid G. (2020). Understanding the effects of sublethal pesticide exposure on honey bees: A role for probiotics as mediators of environmental stress. Front. Ecol. Evol..

[46] Leska A., Nowak A., Motyl I. (2022). Isolation and Some Basic Characteristics of Lactic Acid Bacteria from Honeybee (*Apis mellifera* L.) Environment—A Preliminary Study. Agriculture.

[47] Leska A., Nowak A., Szulc J., Motyl I., Czarnecka-Chrebelska K.H. (2022). Antagonistic Activity of Potentially Probiotic Lactic Acid Bacteria against Honeybee (*Apis mellifera* L.) Pathogens. Pathogens.

[48] Lye H.S., Balakrishnan K., Thiagarajah K., Mohd Ismail N.I., Ooi S.Y. (2016). Beneficial properties of probiotics. Trop. Life Sci. Res..

[49] Chen C.-Y., Tsen H.-Y., Lin C.-L., Lin C.-K., Chuang L.-T., Chen C.-S., Chiang Y.-C. (2013). Enhancement of the immune response against *Salmonella* infection of mice by heat-killed multispecies combinations of lactic acid bacteria. J. Med. Microbiol..

[50] Hafeez A., Iqbal S., Tawab I.A., Bhutto A., Uddin R., Anwar F. (2015). Liquid chromatographic separation and quantification of imidacloprid in different modes of formulations. Am. Eurasian J. Agric. Env. Sci..

[51] Harishankar M.K., Sasikala C., Ramya M. (2012). Efficiency of the intestinal bacteria in the degradation of the toxic pesticide, chlorpyrifos. 3 Biotech.

[52] Trinder M., McDowell T.W., Daisley B.A., Ali S.N., Leong H.S., Sumarah M.W., Reid G. (2016). Probiotic *Lactobacillus rhamnosus* reduces organophosphate pesticide absorption and toxicity to *Drosophila melanogaster*. Appl. Environ. Microbiol..

[53] Wang Y.-S., Wu T.-H., Yang Y., Zhu C.-L., Ding C.-L., Dai C.-C. (2016). Binding and detoxification of chlorpyrifos by lactic acid bacteria on rice straw silage fermentation. J. Environ. Sci. Health B.

[54] Lee J.H., Lee H.Y., Cho D.Y., Kim M.J., Jung J.G., Jeong E.H., Haque M.A., Cho K.M. (2021). Biodegradable properties of organophosphorus insecticides by the potential probiotic *Lactobacillus plantarum* WCP931 with a degrading gene (opdc). Appl. Biol. Chem..

[55] Daisley B.A., Trinder M., McDowell T.W., Welle H., Dube J.S., Ali S.N., Leong H.S., Sumarah M.W., Reid G. (2017). Neonicotinoid-induced pathogen susceptibility is mitigated by *Lactobacillus plantarum* immune stimulation in a *Drosophila melanogaster* model. Sci. Rep..

[56] Sun Z., Sun W., An J., Xu H., Liu Y., Yan C. (2022). Copper and chlorpyrifos stress affect the gut microbiota of chironomid larvae (*Propsilocerus akamusi*). Ecotoxicol. Environ. Saf..

[57] Kumral A., Kumral N.A., Gurbuz O. (2020). Chlorpyrifos and Deltamethrin degradation potentials of two *Lactobacillus plantarum* (Orla-Jensen, 1919) (*Lactobacillales*: *Lactobacillaceae*) strains. Turk. Entomol. Derg..

[58] Pinto G.D.A., Castro I.M., Miguel M.A.L., Koblitz M.G.B. (2019). Lactic acid bacteria–Promising technology for organophosphate degradation in food: A pilot study. LWT.

[59] Cho K.M., Math R.K., Islam S.M., Lim W.J., Hong S.Y., Kim J.M., Yun M.G., Cho J.J., Yun H.D. (2009). Biodegradation of chlorpyrifos by lactic acid bacteria during kimchi fermentation. J. Agric. Food Chem..

[60] Cycoń M., Piotrowska-Seget Z. (2015). Biochemical and microbial soil functioning after application of the insecticide imidacloprid. J. Environ. Sci..

[61] Cycoń M., Markowicz A., Borymski S., Wójcik M., Piotrowska-Seget Z. (2013). Imidacloprid induces changes in the structure, genetic diversity and catabolic activity of soil microbial communities. J. Environ. Manag..

[62] Wang M.-C., Liu Y.-H., Wang Q., Gong M., Hua X.-M., Pang Y.-J., Hu S., Yang Y.-H. (2008). Impacts of methamidophos on the biochemical, catabolic, and genetic characteristics of soil microbial communities. Soil Biol. Biochem..

[63] Đorđević T.M., Šiler-Marinković S.S., Đurović-Pejčev R.D., Dimitrijević-Branković S.I., Gajić Umiljendić J.S. (2013). Dissipation of pirimiphos-methyl during wheat fermentation by *Lactobacillus plantarum*. Lett. Appl. Microbiol..

[64] Jia H.-R., Wu Y.-Y., Dai P.-L., Wang Q., Zhou T. (2015). Effects of the sublethal doses of imidacloprid on the bacterial diversity in the midgut of *Apis mellifera ligustica* (Hymenoptera: Apidae)(*In English*). Acta Entomol. Sin..

[65] Raymann K., Motta E.V., Girard C., Riddington I.M., Dinser J.A., Moran N.A. (2018). Imidacloprid decreases honey bee survival rates but does not affect the gut microbiome. Appl. Environ. Microbiol..

[66] Almasri H., Liberti J., Brunet J.-L., Engel P., Belzunces L.P. (2022). Mild chronic exposure to pesticides alters physiological markers of honey bee health without perturbing the core gut microbiota. Sci. Rep..

[67] Abdou S.M., Abdel-Gawaad A.A., Abo-El-Amaiem E., El-Alfy A.A. (1983). Effect of some organochlorine insecticides on some species of bacteria used in the dairy industry. Egypt J. Dairy Sci..

[68] Alberoni D., Favaro R., Baffoni L., Angeli S., Di Gioia D. (2021). Neonicotinoids in the agroecosystem: In-field long-term assessment on Honeybee colony strength and microbiome. Sci. Total Environ..

[69] Pachla A., Ptaszyńska A.A., Wicha M., Kunat M., Wydrych J., Oleńska E., Małek W. (2021). Insight into probiotic properties of lactic acid bacterial endosymbionts of *Apis mellifera* L. derived from the Polish apiary. Saudi J. Biol. Sci..

[70] Malfatti A., Mallmann G.C., Oliveira Filho L.C., Carniel L.S., Cruz S.P., Klauberg-Filho O. (2021). Ecotoxicological test to assess effects of herbicides on spore germination of *Rhizophagus clarus* and *Gigaspora albida*. Ecotoxicol. Environ. Saf..

[71] Berber İ., Çökmüş C., Atalan E. (2004). Effects of Some Pesticides on Spore Germination and Larvicidal Activity of *Bacillus thuringiensis* var. *israelensis* and *Bacillus sphaericus* 2362 Strain. Turk. J. Biol..

[72] Abou Ayana I.A.A., El Deen A.A., El-Metwall M.A. (2010). Behavior of certain lactic acid bacteria in the presence of pesticides residues. Int. J. Dairy Sci..

[73] Pettis J.S., Collins A.M., Wilbanks R., Feldlaufer M.F. (2004). Effects of coumaphos on queen rearing in the honey bee, *Apis mellifera*. Apidologie.

[74] Haarmann T., Spivak M., Weaver D., Weaver B., Glenn T. (2002). Effects of fluvalinate and coumaphos on queen honey bees (hymenoptera: Apidae) in two commercial queen rearing operations. J. Econ. Entomol..

[75] Yoon K.Y., Woodams E.E., Hang Y.D. (2004). Probiotication of tomato juice by lactic acid bacteria. Korean J. Microbiol..

[76] Zhang M., Ming Y., Guo H., Zhu Y., Yang Y., Chen S., He L., Ao X., Liu A., Zhou K. (2021). Screening of lactic acid bacteria for their capacity to bind cypermethrin in vitro and the binding characteristics and its application. Food Chem..

[77] Rezaei F., Nejati R., Sayadi M., Nematillahi A. (2021). Diazinon reduction in apple juice using probiotic bacteria during fermentation and refrigerated storage. Environ. Sci. Pollut. Res..

[78] Li C., Ma Y., Mi Z., Huo R., Zhou T., Hai H., Kwok L., Sun Z., Chen Y., Zhang H. (2018). Screening for lactobacillus plantarum strains that possess organophosphorus pesticide-degrading activity and metabolomic analysis of phorate degradation. Front. Microbiol..

[79] Yuan S., Li C., Yu H., Xie Y., Guo Y., Yao W. (2021). Selective uptake determines the variation in degradation of organophosphorus pesticides by *Lactobacillus plantarum*. Food Chem..

[80] Sreekumar O., Hosono A. (1998). Antimutagenicity and the influence of physical factors in binding *Lactobacillus gasseri* and *Bifidobacterium longum* cells to amino acid pyrolysates. J. Dairy Sci..

[81] Islam S.M., Math R.K., Cho K.M., Lim W.J., Hong S.Y., Kim J.M., Yun M.G., Cho J.J., Yun H.D. (2010). Organophosphorus hydrolase (OpdB) of *Lactobacillus brevis* WCP902 from kimchi is able to degrade organophosphorus pesticides. J. Agric. Food Chem..

[82] Zhao X.-H., Wang J. (2012). A brief study on the degradation kinetics of seven organophosphorus pesticides in skimmed milk cultured with *Lactobacillus* spp. at 42 °C. Food Chem..

[83] Kostić A., Milinčić D., Petrović T., Krnjaja V., Stanojević S., Barać M., Tešić Ž., Pešić M. (2019). Mycotoxins and mycotoxin producing fungi in pollen: Review. Toxins.

[84] Asurmendi P., Gerbaldo G., Pascual L., Barberis L. (2020). Lactic acid bacteria with promising AFB1 binding properties as an alternative strategy to mitigate contamination on brewers’ grains. J. Environ. Health B.

[85] Genersch E., Gisder S., Hedtke K., Hunter W.B., Möckel N., Müller U. (2013). Standard methods for cell cultures in *Apis mellifera* research. J. Apic. Res..

[86] Panyo J., Matsunami K., Panichayupakaranant P. (2016). Bioassay-guided isolation and evaluation of antimicrobial compounds from *Ixora megalophylla* against some oral pathogens. Pharm. Biol..

[87] Ilboudo S., Fouche E., Rizzati V., Toé A.M., Gamet-Payrastre L., Guissou P.I. (2014). In vitro impact of five pesticides alone or in combination on human intestinal cell line Caco-2. Toxicol. Rep..

[88] Abhishek A., Ansari N.G., Shankhwar S.N., Jain A., Singh V. (2014). In vitro toxicity evaluation of low doses of pesticides in individual and mixed condition on human keratinocyte cell line. Bioinformation.

[89] Abdel-Halim K.Y., Osman S.R. (2020). Cytotoxicity and oxidative stress responses of imidacloprid and glyphosate in human prostate epithelial wpm-Y.1 cell line. J. Toxicol..

[90] Yun X., Huang Q., Rao W., Xiao C., Zhang T., Mao Z., Wan Z. (2017). A comparative assessment of cytotoxicity of commonly used agricultural insecticides to human and insect cells. Ecotoxicol. Environ. Saf..

[91] Pandya N., Thakkar B., Pandya P., Parikh P. (2021). Evaluation of insecticidal potential of organochemicals on SF9 cell line. J. Basic Appl. Zool..

[92] Barabadi H., Najafi M., Samadian H., Azarnezhad A., Vahidi H., Mahjoub M., Koohiyan M., Ahmadi A. (2019). A systematic review of the genotoxicity and Antigenotoxicity of biologically synthesized metallic nanomaterials: Are green nanoparticles safe enough for clinical marketing?. Medicina.

[93] Ren N., Atyah M., Chen W.-Y., Zhou C.-H. (2017). The various aspects of genetic and epigenetic toxicology: Testing methods and clinical applications. J. Transl. Med..

[94] Kızılet H., Yilmaz B., Uysal H. (2019). Herbal Medicine against genotoxicity of dimethoate, an insecticide, in mammalian somatic cells. Heliyon.

[95] Kara M., ÖztaŞ E., Özhan G. (2020). Acetamiprid-induced Cyto- and Genotoxicity in the AR42J Pancreatic Cell Line. Turk. J. Pharm. Sci..

[96] Khodabandeh Z., Etebari M., Aliomrani M. (2021). Study of the probable genotoxic effects of zolone (phosalone) exposure in mice bone marrow derived cells. Genes Environ..

[97] Chandrakar T.R., Singh A.P., Chandra Sarkhel B., Nath Bagchi S. (2020). In vitro cytotoxicity and genotoxicity assessments of carbofuran and malathion pesticides on cat (*Felis catus*) fibroblast cells. Biomed. Pharmacol. J..

[98] Anguiano-Vega G.A., Cazares-Ramirez L.H., Rendon-Von Osten J., Santillan-Sidon A.P., Vazquez-Boucard C.G. (2020). Risk of genotoxic damage in schoolchildren exposed to organochloride pesticides. Sci. Rep..

[99] Saleh M., Din D., Al-Masri A. (2021). In vitro genotoxicity study of the lambda-cyhalothrin insecticide on Sf9 insect cells line using Comet assay. Jordan J. Bio. Sci..

[100] El-Nahhal Y. (2020). Pesticide residues in honey and their potential reproductive toxicity. Sci. Total Environ..

[101] González-Martín M.I., Revilla I., Vivar-Quintana A.M., Betances Salcedo E.V. (2017). Pesticide residues in propolis from Spain and Chile. An approach using near infrared spectroscopy. Talanta.

[102] Friedle C., Wallner K., Rosenkranz P., Martens D., Vetter W. (2021). Pesticide residues in daily bee pollen samples (April-July) from an intensive agricultural region in Southern Germany. Environ. Sci. Pollut. Res. Int..

[103] Villalba A., Maggi M., Ondarza P.M., Szawarski N., Miglioranza K.S.B. (2020). Influence of land use on chlorpyrifos and persistent organic pollutant levels in honey bees, bee bread and honey: Beehive exposure assessment. Sci. Total Environ..

[104] Pareja L., Colazzo M., Pérez-Parada A., Niell S., Carrasco-Letelier L., Besil N., Cesio M.V., Heinzen H. (2011). Detection of Pesticides in Active and Depopulated Beehives in Uruguay. Int. J. Environ. Res. Public Health.

[105] Balayiannis G., Balayiannis P. (2008). Bee Honey as an Environmental Bioindicator of Pesticides’ Occurrence in Six Agricultural Areas of Greece. Arch. Environ. Contam. Toxicol..

[106] Woodcock B.A., Ridding L., Freeman S.N., Pereira M.G., Sleep D., Redhead J., Aston D., Carreck N.L., Shore R.F., Bullock J.M. (2018). Neonicotinoid residues in UK Honey despite European Union moratorium. PLoS ONE.

[107] Valdovinos-Flores C., Gaspar-Ramírez O., Heras-Ramírez M.E., Lara-Álvarez C., Dorantes-Ugalde J.A., Saldaña-Loza L.M. (2016). Boron and Coumaphos residues in hive materials following treatments for the Control of *Aethina tumida* Murray. PLoS ONE.

[108] Tao Y., Dong F., Xu J., Phung D., Liu Q., Li R., Liu X., Wu X., He M., Zheng Y. (2019). Characteristics of neonicotinoid imidacloprid in urine following exposure of humans to orchards in China. Environ. Int..

[109] Mohamed F., Gawarammana I., Robertson T.A., Roberts M.S., Palangasinghe C., Zawahir S., Jayamanne S., Kandasamy J., Eddleston M., Buckley N.A. (2009). Acute human self-poisoning with imidacloprid compound: A neonicotinoid insecticide. PLoS ONE.

[110] Taheri E., Amin M.M., Daniali S.S., Abdollahpour I., Fatehizadeh A., Kelishadi R. (2022). Health risk assessment of exposure to chlorpyrifos in pregnant women using deterministic and probabilistic approaches. PLoS ONE.

